# Changes in gadoxetic-acid-enhanced MR imaging during the first year after irreversible electroporation of malignant hepatic tumors

**DOI:** 10.1371/journal.pone.0242093

**Published:** 2020-11-17

**Authors:** Wolf Bäumler, Andreas Schicho, Jan Schaible, Niklas Verloh, Karin Senk, Phillip Wiggermann, Christian Stroszczynski, Lukas Phillip Beyer

**Affiliations:** 1 Department of Radiology, University Hospital Regensburg, Regensburg, Germany; 2 Department of Radiology and Nuclear Medicine, Hospital Braunschweig, Braunschweig, Germany; 3 Department of Diagnostic and Interventional Radiology, Ernst von Bergmann Hospital, Potsdam, Germany; Medical University of Vienna, AUSTRIA

## Abstract

**Purpose:**

To evaluate the appearance and size of ablation zones in gadoxetic-acid-enhanced magnetic resonance imaging (MRI) during the first year after irreversible electroporation (IRE) of primary or secondary hepatic malignancies and to investigate potential correlations to clinical features.

**Material and methods:**

The MRI-appearance of the ablation area was assessed 1–3 days, 6 weeks, 3 months, 6 months, 9 months and 1 year after IRE. The size of the ablation zone and signal intensities of each follow-up control were compared. Moreover, relationships between clinical features and the MRI-appearance of the ablation area 1–3 days after IRE were analyzed.

**Results:**

The ablation zone size decreased from 5.6 ± 1.4 cm (1–3 days) to 3.7±1.2 cm (1 year). A significant decrease of central hypointensities was observed in T2-blade- (3 months), T2 haste- (6 weeks; 3 months; 6 months; 1 year), T1 arterial phase- (3 months; 1 year), and diffusion-sequences (6 weeks; 3 months; 6 months; 9 months; 1 year). The unenhanced T1-sequences showed significantly increasing central hypointensities (6 weeks; 3 months; 6 months; 9 months; 1 year). Significantly increasing peripheral hypointensities were detected in T1 arterial phase- (3 months; 6 months; 9 months; 1 year) and in T1 portal venous phase-sequences (6 weeks; 3 months; 6 months; 9 months; 1 year). Peripheral hypointensities of unenhanced T1-sequences decreased significantly 1 year after IRE. 1–3 days after IRE central T1 portal venous hypo- or isointensities were detected significantly more often than hyperintensities, if more than 3 IRE electrodes were used.

**Conclusion:**

Hepatic IRE results in continuous reduction of ablation zone size during the first postinterventional year. In addition to centrally decreasing T1-signal and almost steadily increasing signal in the enhanced T2 haste-, diffusion- and T1 arterial phase-sequences, there is a trend toward long-term decreasing T1 arterial- and portal venous MRI-signal intensity of the peripheral ablation area, probably representing a region of reversible electroporation.

## Introduction

Although surgical resection is considered as the most effective treatment method for primary and secondary hepatic malignancies, many patients are not candidates for surgery if the tumor is too advanced or if there are several comorbidities [1]. In these patients, tumor destruction can be achieved by various ablative techniques. However, most of them are based on thermal treatment of the ablated tissue. Thermal damage of adjacent intrahepatic structures, e.g., vessels, can increase the risk of substantial complications [2, 3]. Moreover, the ablation of tumors adjacent to flowing blood can cause a so called "heat sink effect", which can affect the size of the ablation area [4], potentially leading to insufficient ablation. Irreversible electroporation (IRE), as a predominantly nonthermal ablative method, represents a viable alternative to thermal ablations. Because it causes cell death through the repeated application of high-voltage electrical impulses that create irreversible damages to the membranes of tumor cells [5], IRE does not have limitations such as those associated with thermal ablation. Consequently, IRE has become more important in the therapy of primary and secondary hepatic tumors in the last few years. As magnetic resonance imaging (MRI) is considered the reference standard for the detection of hepatic tumors [6, 7], it is very important that radiologists are aware of all the different appearances of the ablation area to be able to differentiate post-ablative tissue from local tumor recurrence. The current literature provides little information about the appearance of the ablation zone in gadoxetic-acid-based MRI after IRE of hepatic malignancies. In particular, long-term data are hard to find. The aim of this study was to evaluate the appearance and the size of the ablation zone in gadoxetic-acid-based MRI during the first year after undergoing IRE of primary or secondary hepatic malignancies. Moreover, the relationship between the MRI appearance of the ablation area 1–3 days after IRE and several clinical features were analyzed.

## Material and methods

### Study design, participant selection and patient characteristics

MRI images of all ablations performed at University Hospital Regensburg between December 2011 and June 2018 were retrospectively evaluated. To investigate the MR imaging appearance of the ablation area after percutaneous IRE of malignant liver tumors, the single-center retrospective observational study was approved by the local ethics committee (Ethics Committee of the University of Regensburg approval number 18-1027-104) and followed the guidelines outlined in the Declaration of Helsinki. The following inclusion criteria were applied to participate in the study: (I) Histologically proven primary or secondary liver malignancies; (II) liver tumors treated by percutaneous IRE; (III) examination by contrast-enhanced MR images of the entire liver before the intervention and at 6 defined times after IRE: 1–3 days, 6 weeks, 3 months, 6 months, 9 months, and 1 year after the ablation; (IV) written informed consent was obtained from the patient for the acquisition of pre- and postinterventional contrast-enhanced MR images, the ablation procedure, and the anonymous use of the data for scientific purposes. All collected data were immediately anonymized after the intervention. Analysis was only started after the data was anonymized. Patients were considered for participating in the current study if all the mentioned criteria (I-IV) were completely satisfied. All the other patients were excluded. In total, 117 patients (86 men and 31 women) fulfilled the inclusion criteria. In 32 of 117 patients, a residual tumor (n = 9) was detected 1–3 days after IRE or local tumor recurrence (n = 23) could be proven within one year after the intervention. These patients were also excluded, because an objective evaluation of the MRI appearance of the ablation area would not have been possible because of the signal behavior of the tumor tissue. Finally, 85 patients were included in the study. In each patient, the ablation of one tumor lesion was performed. Additionally, serum bilirubin, alkaline phosphatase, aspartate aminotransferase and alanine aminotransferase levels were measured preinterventionally and at each of the defined follow-up controls after IRE. The patient and disease characteristics are listed in Table 1.

**Table 1:** Baseline patient and disease characteristics.

Characteristics	
Age (y)	
Mean ± SD	67.4 ±11.0
Range	36–85
Sex, n (%)	
Male	66 (77.6)
Female	19 (22.4)
Tumor diameter (cm)	
Mean ± SD	2.3 ± 1.1
Range	0.4–4.4
Patients with liver cirrhosis, n (%)	36 (42.4)
Tumor localization, n (%)	
Segment I	5 (5.9)
Segment II	9 (10.6)
Segment III	9 (10.6)
Segment IVa	8 (9.4)
Segment IVb	10 (11.8)
Segment V	11 (12.9)
Segment VI	10 (11.8)
Segment VII	7 (8.2)
Segment VIII	16 (18.8)

### Irreversible electroporation (IRE)

All IREs were performed percutaneously under computed tomographic fluoroscopy guidance and full anesthesia including deep muscle relaxation using the NanoKnife system (AngioDynamics, Latham, New York). The operator installed two to six monopolar 18-gauge IRE probes parallel to each other in or around the target tumor. The IRE parameters were as follows: pulses per cycle, 70; pulse length, 90 μs; electric field, 1500 V/cm needle distance. A test pulse of 270 V was delivered before the delivery of the 90 therapeutic pulses to confirm sufficient conductivity.

### Image acquisition

Every patient was examined by contrast-enhanced MR scans using the hepatocyte-specific contrast agent gadolinium ethoxybenzyl diethylenetriamine pentaacetic acid (Primovist; Bayer Schering, Berlin, Germany). MR imaging was performed with a clinical whole-body 3-T system (MAGNETOM Skyra; Siemens, Erlangen, Germany). The contrast agent was administered by intravenous bolus injection at a flow rate of 1 mL/s and flushed with 20 mL of NaCl 0.9% and its dose was adapted according to the patient´s body weight (0.025 mmol/kg of body weight).

Every sequence contained the entire liver. The MR protocol included the following sequences: T2 haste contrast-enhanced, T1 vibe3d fat suppressed unenhanced, T1 vibe3d fat suppressed contrast-enhanced arterial phase, T1 vibe3d fat suppressed contrast-enhanced portal venous phase, T2 blade fat suppressed contrast-enhanced, diffusion trace contrast-enhanced (b-value: 800 s/mm^2^) and T1 vibe3d fat suppressed delayed phase. Both the pre- and postinterventional MR images were analyzed by two radiologists with 4 and 8 years of experience in abdominal imaging. The appearance of the ablation zone in each MR image was examined for at the defined points of time by consensus reading. The ablated area was classified into a central and a peripheral zone, which were evaluated separately from each other. The signal intensity was evaluated in each sequence and categorized in three groups: hypointense, isointense, or hyperintense relative to the healthy liver parenchyma in the same sequence. Moreover, the size of the postablative area was assessed in the T1 vibe3d fat suppressed delayed phase by using the axial image with the largest ablation dimension.

### Statistical analysis

All collected data are presented as frequency counts and percentages. To identify significant changes in the size of the ablation area, a single factor variance analysis with repeated measurement (ANOVA) was used. As effect estimates, 95% confidence intervals are presented. A p-value of ≤ 0.05 was considered statistically significant in all statistical analyses. The McNemar-test was applied for the identification of significant differences (hypointense vs. isointense/hyperintense signal) between signal intensities 1–3 days after IRE and each of the subsequent follow-up controls. A binary logistic regression model was used to identify potential correlation between clinical features and the MRI appearance (hypointense or isointense vs. hyperintense signal) of the ablation area 1–3 days after IRE. Equal correlation of the binary response for individual patients was assumed, implying an exchangeable correlation structure. The analyzed variables were age (<65 years vs. ≥65 years), sex, the presence of liver cirrhosis, the size of the ablation area (≤ 4 cm vs > 4 cm), tumor type (primary vs. secondary hepatic malignancy), and the number of IRE electrodes (≤3 vs. >3). All statistical analyses were performed with SPSS statistics (IBM SPSS Statistics, version 25).

## Results

**Fig 1:**
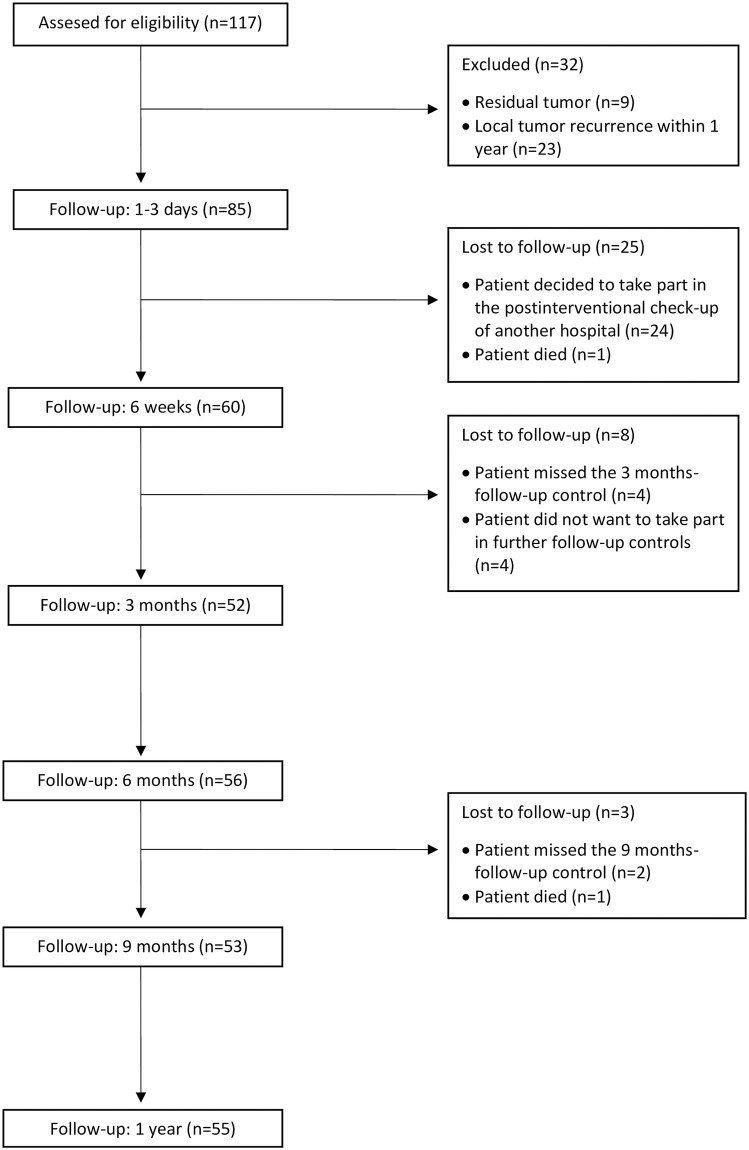
Number of patients during follow-up. The left column shows the numbers of patients at each point of follow-up. The reasons for an exclusion or a lost to follow-up are presented in the right column.

The complete number of follow-up controls was generated for 52 of 85 patients. The number of patients and the different reasons they were lost to follow-up during the first year after IRE are indicated in Fig 1. An overview of the different tumor types is presented in Table 2. 53 patients suffered from hepatocellular carcinoma, 5 from cholangiocellular carcinoma. 27 patients presented hepatic metastasis that originated from colorectal carcinoma (n = 21), mammarian carcinoma (n = 3), carcinoma of unknown origin (n = 1), choroidal melanoma (n = 1) and pancreatic adenocarcinoma (n = 1). In 85 patients suffering from hepatic malignancies, the mean tumor diameter was 2.3 ± 1.1 cm (Table 1).

**Table 2:** Tumor types of 85 patients treated with irreversible electroporation of malignant liver tumors.

Diagnosis	Number of patients
*Primary liver tumors*	
Hepatocellular carcinoma	53
Cholangiocellular carcinoma	5
*Metastasis of*	
Colorectal tumor	21
Mammarian carcinoma	3
Carcinoma of unknown origin	1
Choroidal melanoma	1
Pancreatic adenocarcinoma	1
**Total**	**85**

The mean diameter of the ablation area 1–3 days after IRE was 5.6 ± 1.4 cm and decreased continuously up to 3.7 ± 2.1 cm at the last follow-up control 1 year after the intervention, corresponding to a reduction in size of 33.9%. The largest decrease in ablation size was detected between the first 3 days and 6 weeks after IRE. A significant change in the ablation size was evident at the second and third follow-up control (Table 3).

**Table 3:** Characteristics of the ablation area during the follow-up.

Point of time	Diameter of the ablation area (cm), mean ± SD	P Value*	95% CI*
**1–3 days after IRE**	5.6 ± 1.4		
**6 weeks after IRE**	4.4 ± 1.5	0.013	0.91–1.32
**3 months after IRE**	4.1 ± 1.4	0.018	0.05–0.96
**6 months after IRE**	3.8 ± 1.6	1.000	- 0.29–0.70
**9 months after IRE**	3.8 ± 2.0	1.000	- 0.72–0.45
**1 year after IRE**	3.7 ± 2.1	1.000	- 0.69–0.54

SD, standard deviation; CI, confidence interval.

*P value and confidence interval concerning the changes of the diameter of the ablation area compared to the previous follow-up control.

A summary of the evolution of laboratory values is presented in Table 4. Elevated laboratory levels were observed preinterventionally in alkaline phosphatase, aspartate aminotransferase and alanine aminotransferase. A postinterventional elevation of the observed laboratory values was detected up to 6 months after IRE, except for alkaline phosphatase, which showed a continuous elevation during the first postinterventional year.

**Table 4:** Evolution of laboratory values during the first year after IRE in 85 patients.

	Pre-IRE	Day 1–3	6 weeks	3 months	6 months	9 months	1 year
Serum Bilirubin (U/L)	0.8	1.7	1.0	1.2	1.1	1.0	0.9
Alkaline phosphatase (U/L)	126.5	141.3	136.4	143.5	132.2	128.7	122.4
Aspartate aminotransferase (U/L)	65	172	90	70	62	48	49
Alanine aminotransferase (U/L)	67	133	83	69	53	47	43

IRE, Irreversible Electroporation; Standard value of serum bilirubin: 0.2–1.0 mg/dL; Standard value of alkaline phosphatase: 45–117 U/L; Standard value of aspartate aminotransferase ≤ 50 U/L; Standard value of alanine aminotransferase ≤ 50 U/L.

**Fig 2:**
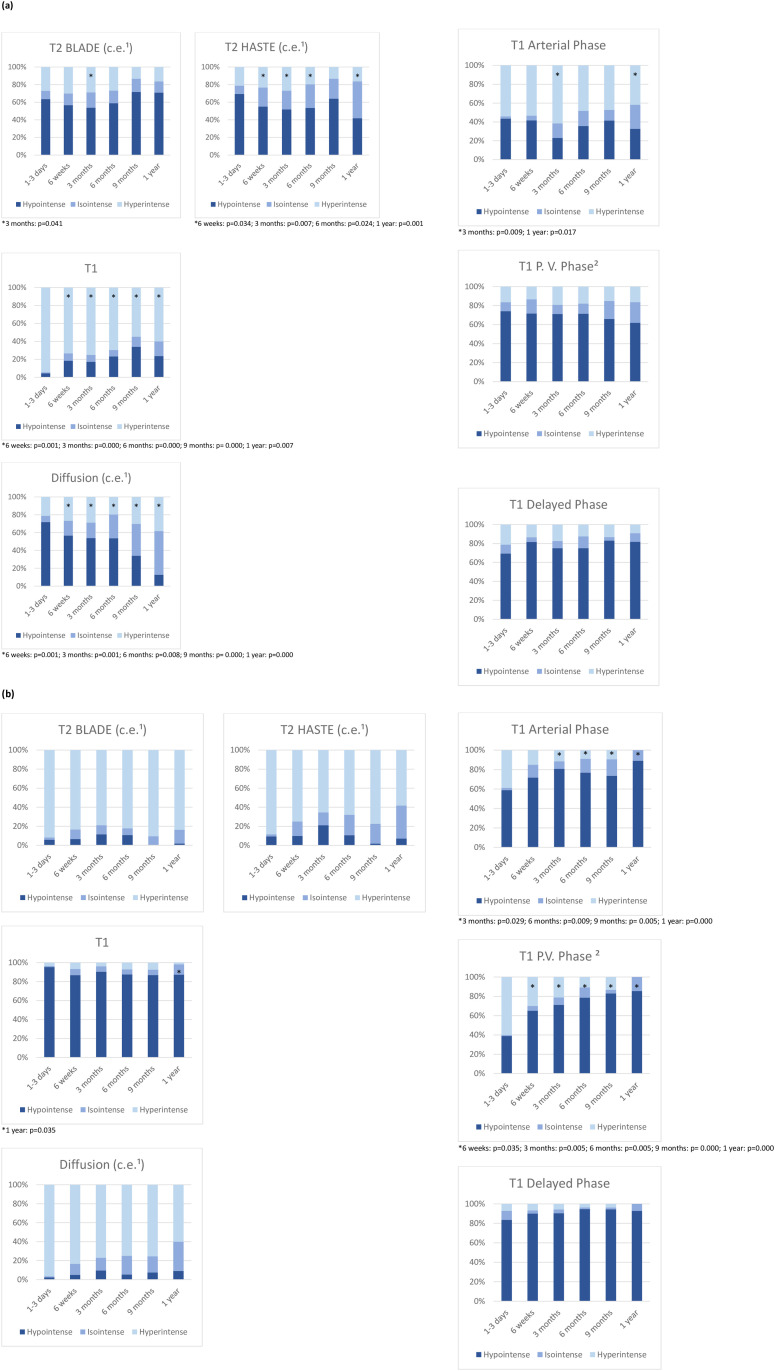
MR imaging appearance after IRE of a HCC located in segment I of a 58-year old man. (a) Postinterventional (1 day after IRE) non-enhanced T1 vibe 3d fat suppressed magnetic resonance imaging shows an ablation area with a hypointense rim (white arrow) and a hyperintense center (red arrow). (b) 1 day after IRE the ablation area shows a hyperintense rim (white arrow) and a hypointense central zone (red arrow) in the contrast-enhanced T2 haste-sequences.

**Fig 3:**
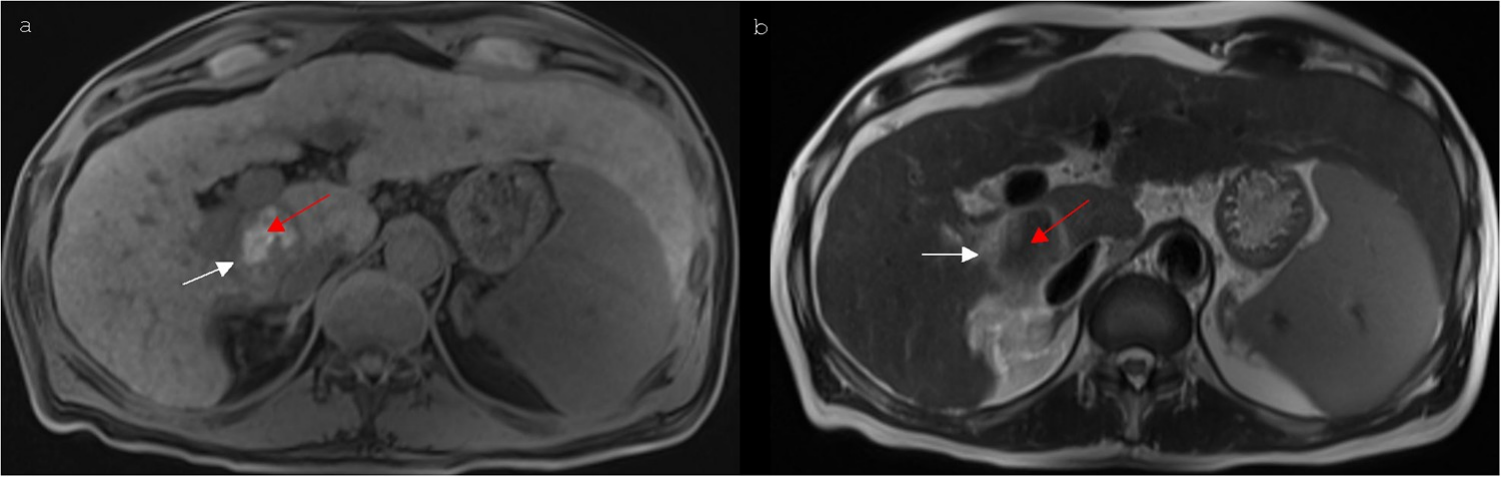
MR imaging appearance after IRE of a HCC located in segment IVb of a 75-year old man. (a) Postinterventional (1 day after IRE) T1 vibe3d fat suppressed contrast-enhanced MRI obtained in the portal venous phase shows an ablation area with a lot of hyperintense parts in its peripheral zone (red arrow) largely regressing up to the 6-weeks follow-up control (b).

**Fig 4:**
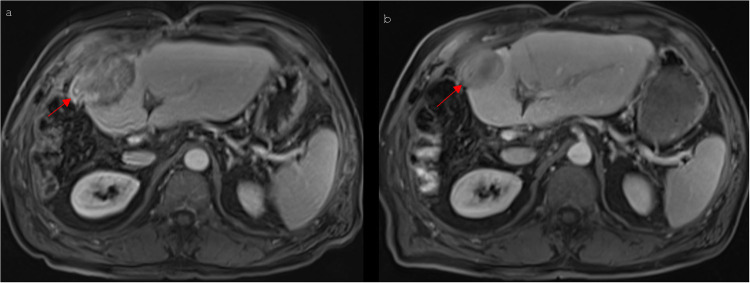
Signal intensity of the ablation zone over time. Different MR images obtained in the center (a) and in the peripheral zone (b) of the ablation area (c.e.^1^: contrast-enhanced; P.V.^2^: portal venous). Significant differences during the follow-up are highlighted (*).

In the majority of the cases, the center of the ablated area showed a mainly hypointense signal in the T2 blade-, T2 haste-, T1 portal venous phase- and T1 delayed phase-sequences, whereas a hyperintense signal was predominantly detected in the unenhanced T1- (Fig 2) and the T1 arterial phase-sequences. The predominant hypointense signal of the diffusion-weighted sequences, which could be recognized in the center of the ablation area 1–3 days after IRE, showed a progressive decrease during the first year after IRE (6 weeks: p = 0.001; 3 months: p = 0.001; 6 months: p = 0.008; 9 months: p = 0.000; 1 year: p = 0.000). Moreover, a significant regression of central hypointense signal intensity was observed during the 3 months follow-up control in the T2-blade-sequences (p = 0.041), 6 weeks (p = 0.034), 3 months (p = 0.007), 6 months (p = 0.024) and 1 year (p = 0.001) after IRE in the T2 haste-sequences, and during the 3 months- and 1 year follow-up control in the T1 arterial phase-sequences (3 months: p = 0.009; 1 year: p = 0.017). The unenhanced T1-sequences showed a significantly increasing central hypointensity from the 6 weeks up to the 1 year follow-up control (6 weeks: p = 0.001; 3 months: p = 0.000; 6 months: p = 0.000; 9 months: p = 0.000; 1 year: p = 0.007). In most cases the rim of the ablation zone appeared predominantly hypointense in the T1-, T1 arterial phase- and the T1 delayed phase-sequences, whereas a primarily peripheral hyperintense signal was noted in the T2 blade-, T2 haste- (Fig 2) and the diffusion-weighted sequences. The hypointense signal, which was predominantly observed 1–3 days after IRE in the peripheral ablation zone of the T1 portal venous phase-sequences, increased significantly during the observed period of time (6 weeks: p = 0.035; 3 months: p = 0.005; 6 months: p = 0.005; 9 months: p = 0.000; 1 year: p = 0.000), Fig 3. A significant increase of peripheral hypointense signal intensity could also be noted in the T1 arterial phase-sequences from the 3 months- up to the 1 year follow-up control (3 months: p = 0.029; 6 months: p = 0.009; 9 months: p = 0.005; 1 year: p = 0.000). Furthermore, the predominant peripheral hypointense signal of the unenhanced T1-sequences decreased significantly 1 year after IRE (p = 0.035). The mean percentage changes in each dimension are shown in Fig 4.

In the binary logistic regression model the application of more than 3 IRE electrodes correlated significantly (p = 0.037) with a hypointense or isointense MRI appearance of the central ablation area in the T1 portal venous phase-sequences 1–3 days after IRE (Table 5).

**Table 5:** Results of binary logistic regression model predicting hypointense or isointense MRI appearance of the center (a) and the peripheral zone (b) of the ablation area.

	T2 Blade	T2 HASTE*	T1	Diffusion*	T1 Arterial Phase	T1 P. V. Phase	T1 Delayed Phase
	P-Value	P-Value	P-Value	P-Value	P-Value	P-Value	P-Value
**(a)**							
Age: < 65y vs ≥65y	0.364	0.793	0.873	0.730	0.587	0.081	0.181
Sex: female vs male	0.764	0.959	0.999	0.613	0.547	0.134	0.254
Liver cirrhosis	0.885	0.379	0.999	0.408	0.806	0.855	0.766
Size of the ablation area: ≤ 4 cm vs > 4 cm	0.472	0.174	0.998	0.162	0.458	0.114	0.863
Tumor type: primary vs secondary malignancies	0.871	0.438	0.999	0.135	0.570	0.977	0.872
Number of IRE electrodes: ≤ 3 vs > 3	0.369	0.767	0.999	0.827	0.450	0.037*	0.271
**(b)**							
Age: < 65y vs≥65y	0.254	0.115	0.998	0.997	0.727	0.351	0.765
Sex: female vs male	0.922	0.141	0.999	0.998	0.321	0.777	0.999
Liver cirrhosis	0.153	0.149	1.000	0.997	0.576	0.212	0.826
Size of the ablation area: ≤ 4 cm vs > 4 cm	0.874	0.930	0.998	1.000	0.129	0.085	0.998
Tumor type: primary vs secondary malignancies	0.270	0.082	1.000	0.998	0.646	0.845	0.998
Number of IRE electrodes: ≤ 3 vs > 3	0.999	0.999	0.999	0.999	0.359	0.449	0.999

*Significant changes

## Discussion

IRE, as a predominantly nonthermal ablative method, has become increasingly important in the therapy of primary and secondary hepatic tumors. Although several reports concerning the safety and efficacy of hepatic IRE have been published in recent years [8–12], not much is known about the appearance of the ablation zone in postinterventional MRI. In particular, long-term data of gadoxetic, to our knowledge, are not existent at the moment. In our opinion, the knowledge of typical MRI appearance is very important to avoid diagnostic errors and to accurately detect residual tumor or local tumor recurrence in the follow-up of patients after IRE.

Although the current study on 85 patients presents a broad spectrum of different MRI signal intensities and a huge disparity in size of the ablated area during the first year after IRE, in most of the patients, the following pattern could be noted:

A continuous decrease in the size of the ablation area was observed up to the 1-year follow-up control, which was assessed in the T1 delayed phase-sequence because it reveals the highest correlation with the pathologic ablation zone size [13]; this was consistent with the results reported by other authors [14, 15]. Similar to the observations of Padia et al., who evaluated the gadodiamide-enhanced MRI of 20 patients with HCC for 300 days after IRE and reported the largest reduction of the ablation size during the first month after the intervention [14], the greatest decrease of the ablation zone was noted between 1–3 days and 6 weeks after IRE with a reduction of its diameter from 5.6 cm to 4.4 cm (Table 3).

In the contrast-enhanced T2 blade- and the contrast-enhanced T2 haste-sequences, most of the observed ablation areas showed a central hypointensity and a peripheral hyperintensity, similar to the findings of Barabasch et al. [15], who depicted an equivalent signal behavior in the T2-weighted sequences during the first postinterventional 8 weeks evaluating 27 patients with 37 liver metastasis for at least 1 year after IRE by gadobutrol-enhanced MRI. Furthermore, the authors reported a complete healing of the ablation zone in 57% [15] of cases, which is not in accordance with our results; in the current study, all the ablation zones were visible at the 1-year follow-up control. The center of the ablation area mainly appeared hyperintense in the unenhanced T1-sequences, whereas its peripheral zone presented a mostly hypointense signal, which was consistent with the observations of Padia et al. [14] and Granata et al. [16], who assessed the gadoxetic-acid-based MRI of 20 patients with HCC before and 1 month after IRE. The gadoxetic-acid-enhanced T1-sequences showed a primarily hypointense signal during the first year after IRE except the central part of the ablation zone in the T1 arterial phase-sequences and particularly the peripheral zone of the ablation area in the T1 portal venous phase-sequences 1–3 days after IRE, in which a rapid increase in the hypointense regions was observed (Fig 4). Besides the observed increase of central hypointense signals in the unenhanced T1-sequences during follow-up and the mostly continuous decrease of central signal intensities in the T2-haste, diffusion- and T1-arterial phase-sequences, overall, a significant trend toward long-term decreasing signal intensity of arterial and especially portal venous T1-sequences was observed in the peripheral ablation zone. In a study by Breen et al., which focused on radiofrequency thermal ablation, imaging findings and tissue necrosis have been correlated: The hyperintensity of the peripheral ablation zone detected in gadolinium contrast-enhanced MRI immediately after IRE was in accordance with necrosis having been proven in histologic analysis [17]. Similar to the findings of Padia et al. [14], the peripheral hyperintense region of the contrast enhanced T1-sequences decreased during the follow-up, which were progressively substituted by normal-appearing liver parenchyma. Consequently, there is a doubt whether the postinterventional peripheral hyperintense region observed in some cases is induced by tissue necrosis. Instead, the mentioned effect could more likely be caused by resolution of the reversible penumbra in the peripheral ablation zone, within the meaning of a reversible electroporation, as previously proposed in the literature [14]. Because in most of the patients the indication for IRE was represented by the fact that they were not candidates for surgical resection because of their general state of health, an additional correlation with tissue samples acquired by biopsy was relinquished. A significant decrease of the hypointense signals was detected in the central ablation area in the contrast-enhanced diffusion weighted sequences, whereas no significant changes of the hypointense signal intensities were observed in the periphery of the ablation zone. However, in the peripheral ablation zone of diffusion weighted sequences there was a trend toward more isointense regions over time (Fig 4), with most of the peripheral regions appearing hyperintense. The mentioned temporary changes of the signal intensity in contrast-enhanced diffusion-weighted sequences can be caused by local cytotoxic edema [15], local vascular dilatation or congestion, or inflammatory processes, as reported in animal studies [18].

A hypointense or isointense signal of the central ablation area in the T1 portal venous phase-sequences occurred significantly more frequent than a hyperintense signal 1–3 days after IRE if more than 3 IRE electrodes were used (p = 0.037). This result indicates that variable methods of IRE seem to cause certain effects in the appearance of the MRI image of the ablation zone, which should be paid more attention in future studies.

The current study has several limitations: The first one is the retrospective nature of the study. The second limitation is the incomplete data acquisition during the follow-up of 33 patients, especially the large number of patients lost to follow-up between day 1–3 and 6 weeks after IRE must be emphasized. Third, the present study cannot draw an exact distinction between necrotic and non-necrotic tissue, as histologic evaluation was challenging in the patient population. Fourth, the study group consisted of a heterogenous patient population in terms of sex, tumor type and liver cirrhosis.

## Conclusions

The current study suggests that there is a continuous decrease in the mean diameter of the ablation area during the first year after IRE. In addition to decreasing unenhanced T1-signal and almost steadily increasing signal in the enhanced T2 haste-, diffusion- and T1 arterial phase-sequences of the central ablation zone, a trend toward long-term decreasing T1 arterial and portal venous signal intensity of the peripheral ablation area can be observed in post-procedure MRI, probably representing resolution of reversible penumbra. The use of more than 3 IRE electrodes significantly influences the MRI appearance of the central ablation zone in the T1 portal venous phase-sequences 1–3 days after IRE.

## Supporting information

S1 Material (SAV)

## Abbreviations

CI: Confidence interval
e.g.: For example
HCC: Hepatocellular carcinoma
IRE: Percutaneous irreversible electroporation
MRI: Magnetic resonance imaging
SD: Standard deviation
